# The impact of nurse practitioners on care delivery in the emergency department: a multiple perspectives qualitative study

**DOI:** 10.1186/1472-6963-13-356

**Published:** 2013-09-22

**Authors:** Julie Li, Johanna Westbrook, Joanne Callen, Andrew Georgiou, Jeffrey Braithwaite

**Affiliations:** 1 Centre for Health Systems and Safety Research, Australian Institute of Health Innovation, Faculty of Medicine, University of New South Wales, Kensington, NSW 2052, Australia; 2 Centre for Clinical Governance Research, Australian Institute of Health Innovation, Faculty of Medicine, University of New South Wales, Kensington, NSW 2052, Australia

## Abstract

**Background:**

Despite well-articulated benefits, the introduction of Nurse Practitioners (NPs) in Australia has been slow. Poorly defined nomenclature relating to advanced practice roles in nursing and variations in such roles both across Australia and worldwide have resulted in confusion and uncertainty regarding the functions and roles of NPs. Qualitative studies focussing on the perceived impact on the care settings into which NPs are introduced are scarce, but are valuable in providing a complete contextual account of NPs in care delivery settings. This study aimed to investigate the perceived impact of the NP on the delivery of care in the ED by senior doctors, nurses, and NPs. Results will facilitate adoption and best use of this human resource innovation.

**Methods:**

A cross-sectional qualitative study was undertaken in the Emergency Departments (EDs) of two large Australian metropolitan public teaching hospitals. Semi-structured, in-depth interviews were conducted with five nurse practitioners, four senior doctors (staff specialists and ED directors) and five senior nurses. Transcribed interviews were analysed using a grounded theory approach to develop themes in relation to the conceptualisation of the impact of the NP role on the ED. Member checking of results was conducted by revisiting the sites to clarify findings with participants and further explore emergent themes.

**Results:**

The impact of the NP role was perceived differently by different groups of participants. Whilst NPs were observed to deliver few quantitative improvements to ED functioning from the perspective of ED directors, NPs believed that they assisted doctors in managing the increasing subacute presentations to the contemporary ED. NPs also believed they embraced a preventative paradigm of care which addressed the long term priorities of chronic disease prevention and cost containment in the broader healthcare environment. The ambiguous position of the NP role, which crosses the gap between nursing and medicine, emerged and resulted in a duality of NP governance.

**Conclusions:**

Interpretation of the NPs’ role occurred through different frames of reference. This has implications for the development of the NP role in the ED. Collaboration and dialogue between various stakeholders, such as ED doctors and senior nursing management is required.

## Background

Originating in the 1960s in the United States, Nurse Practitioners (NPs) are advanced practice nurses who have completed additional training beyond that of a registered nurse [1,2]. The NP role has continued to develop and they now work in a range of health care settings in the US and other countries including Canada and the United Kingdom (UK). NPs provide an alternative source of care to General Practitioner services, with a focus on health promotion and disease prevention, education, and encouraging increased patient participation and decision making in the care process [3-6]. The NP role was first introduced in the state of New South Wales (NSW), Australia in 1990. NPs in Australia are senior Registered Nurses who have completed a University awarded Masters Degree. NPs in Australia perform advanced clinical roles involving the autonomous management of entire patient episodes which fall within a defined scope of practice. Their roles are predicated on bridging gaps between medicine and nursing, addressing deficiencies in access, efficiency and quality of services and under-serviced demographic groups where the maldistribution of medical practitioners geographically and across specialties lead to significantly discrepant levels of healthcare access [7]. NPs have also been trained to work in specialist services in acute care areas, including emergency departments and intensive care units [8,9]. Variations in the roles that NPs adopt, both nationally and internationally, have been found [10-13]. The implementation of NPs in Australia has been mitigated by role ambiguity, confusion and uncertainty amongst both health care professionals and consumers in relation to their function and role [10-13].

Qualitative studies focussing on clinician perceptions and understandings of the NP role, as well as the perceived impact on the care settings into which they are introduced, are scarce. These types of studies are necessary because they add information beyond identifying and quantifying the prevalence of a particular view. One qualitative study which entailed interviews with nine key stakeholders undertaken by Tye [14] in a London hospital identified organisational factors such as consensus about the NP scope of practice, among others, as contextual mechanisms which play a role in facilitating successful NP implementation.

There are grounds for believing that different professional perspectives are indicators of distinguishable frames of reference. A frame of reference is “a built-up repertoire of tacit knowledge that is used to impose structure upon and impart meaning to otherwise ambiguous social and situational information to facilitate understanding” [15]. Frames of reference serve as implicit guidelines which organise and shape individual interpretations of events and meanings of contextual phenomena, and are shaped and constrained by an individual or group’s purpose, context, power or knowledge base [16,17]. Orlikowski and colleagues posited that degrees of alignment between organisational members’ assumptions, expectations, interpretations and understanding of the role of an innovation ultimately determined outcomes following implementation [18]. Incongruent frames lead to difficulties and conflicts around developing, appropriation, and use of the innovation [18]. Perceptions and attitudes towards the role of NPs by other members of the clinical team are likely to contribute to the extent to which NPs are able to successfully fulfil their role [19]. This study sought to investigate the perceptions of senior doctors and nurses, and NPs, to provide insight on the extent to which there is consistency between the promoted benefits of the NP role on care delivery and the perceptions of staff about these issues. We chose an ED setting because it offered the potential to appreciate attitudes to NPs in a dynamic, time-critical environment which could shed light on their roles.

## Methods

### Research question

What do senior doctors, nurses, and NPs perceive to be the impact of NPs on the delivery of care in the ED?

### Design and setting

A qualitative study using in-depth semi-structured interviews of NPs, senior doctors and senior nurses from Emergency Departments in two large Australian metropolitan public teaching hospitals was undertaken (Table 1). Qualitative research methods are effective for in-depth explorations of clinician perceptions regarding roles and clinical work practice [20,21]. The hospitals were located within the same geographic Area Health Service under a centralised administrative structure. This study is part of a broader study which explored the way NPs integrate ICT into their work [22]. The NP role had been introduced at both hospitals approximately four years prior to data collection and is reported in the previous paper [22]. Ethics approval for the study was granted by the relevant Area Health Service Research Ethics Committee and Human Research Ethics Committees of both hospitals and the University of New South Wales.

**Table 1:** Characteristics of study sites and participants

Characteristics*	**Hospital site**	
	**A**	**B**
Hospital Beds	445	758
Annual Discharges	29,939	83,898
Annual ED Attendances	51,105	61,939
Annual ED Discharges	40,323	40,713
ED Interviews		
Nurse Practitioners (n = 5)	4 Nurse Practitioners	1 Nurse Practitioner
Senior Doctors (n = 4)	ED Director^#^	ED Director^#^
	1 senior Staff Specialist	1 senior Staff Specialist
Senior Nurses (n = 5)	1 Nurse Unit Manager	2 Nurse Unit Managers
	1 Advanced Clinical Practice Nurse	1 senior registered nurse

*Hospital statistics over year 2009/2010.

^#^In Australia, ED Directors engage in direct patient care during clinical shifts in addition to general departmental management.

### Participant selection and sampling

Fourteen semi-structured interviews (Table 2) were undertaken with five nurse practitioners, four senior doctors (two ED Directors and two senior staff specialists) and five senior nurses (three Nurse Unit Managers, an Advanced Clinical Practice Nurse and a senior Registered Nurse) between July 2010 and January 2011. A prominent outcome of NP implementation in Australia is the resulting discordance between what the role does and is perceived to entail, across different care settings and between different clinical groups and consumers [11,12]. A pilot interview with one NP participant at Hospital Site A revealed similar issues as those raised in the research [11,12]. Therefore, lead interview questions (Table 2) were informed by the literature and the pilot interview as well as by data obtained during direct observation of two nurse practitioners at Hospital Site A.

**Table 2:** Lead questions guiding semi-structured interviews with emergency department doctors, nurses and NPs

**Questions for nurse practitioners**	**Questions for senior doctors and nurses**
1. How would you describe your role?	1. What is your understanding of the role of the NP?
2. How is the role of a Nurse Practitioner (NP) different from that of doctors and other nurses?	2. What do you perceive is the impact of the NP role on the delivery of care?
− What is the impact of the NP on the delivery of care?	3. How do you think the role of the NP influences the roles of doctors and nurses?
3. Do you think that the roles of existing Emergency Department (ED) clinicians have changed as a result of the NP role?	4. What are the mechanisms that determine the role of the NP?
4. Do you think the roles of NPs would differ at other sites?	5. What is your view on the overlap between NP roles and those of doctors?
− Why do you think they would differ? (e.g. due to ICT, casemix, culture etc.)	
− What are the mechanisms which determine the role of the NP? For example, what factors lead to these differences across sites?	
5. What is your view on the overlap between NP roles and those of doctors?	

Interviews undertaken were semi-structured with no pre-coded response categories. The interview questions were designed to introduce a broad theme for exploration and discussion with the participant. Questions were phrased differently to take account of the different clinical roles of those being interviewed [23], however they essentially addressed the same thematic content. All NPs (n = 5) employed at the two EDs were interviewed whilst doctors and nurses were purposively selected based on seniority of role and length of employment at the sites to ensure they had knowledge of the development of the NP role onsite and had reasonable levels of interaction with NPs during clinical work.

A document outlining the study, its voluntary nature, confidentiality of results and participants, and a consent form were witnessed and signed by participants. An opportunity to raise queries related to the study was also presented at this time. Participants were recruited via email and approached personally in the department when email contact failed. Interviews with senior doctors and nurses regarding their understanding of the NP role were triangulated with data from NPs.

### Data analysis

A grounded theory approach using thematic content analysis was employed to derive themes. Grounded theory refers to the process of inductively developing theory from systematically gathered and analysed data [24]. Inductive theory-building is especially relevant to observational studies where conditions under which phenomena are observed cannot be controlled [23]. Interview transcripts were analysed inductively over a multi-phase process. Interview questions were employed in the initial stage of analysis to form broad, macro-codes and a basic taxonomy under which open-coded content could be classified. Portions of transcribed interview text relating to each question were analysed by one researcher (JL) to develop lists of specific codes representing the various attributes and dimensions of each macro-code. Transcript extracts were matched with corresponding codes. Electronic management and coding of data using NVivo (v.8) software ensured automatic collation of all data extracts within each code. Coding reached theoretical saturation when no new themes emerged. Axial coding of related data extracts within and across categories was undertaken in a second phase to identify meaningful relationships between codes and higher level, recurring themes. Revision and finalisation of themes was achieved via triangulation and consensus between the research team. Member checking of results occurred through follow up interviews with relevant participants to clarify findings with participants and further explore emergent themes.

## Results

### Impact on ED functioning

NPs, senior doctors and senior nurses perceived a range of changes to the overall functioning of the ED following the introduction of NPs (Table 3). Nurse Unit Managers (NUMs) noticed significant efficiencies in patient throughput when NPs contributed to patient management on the ED floor. This was particularly important in relieving waiting room queues for patients presenting with minor complaints and assisted the department in meeting hospital Key Performance Indicators which recommended desired patient wait-time limits. Senior doctors and nurses also agreed that NPs relieved the subacute workload of doctors and facilitated their availability for acute presentations where their advanced medical expertise was better utilised. NPs expressed the view that they addressed a significant change in the nature of emergency care brought about by changing consumer needs; in addition to providing emergency care, NPs described the contemporary ED as a “one stop shop” which addresses certain care services which may be lacking in the primary care setting. Examples included after-hours care and simple procedures such as suturing and plastering which may not be offered at local GP practices. NPs felt that advanced practice nurses were the ideal alternative to doctors for addressing the increasing number of subacute patients.

**Table 3:** Perceived impact of NPs on care delivery in the emergency department– representative quotes

**Category**	**Quotation**
Impact on ED Functioning	“working in here, the Nurse Practitioner sees a lot of patients and we see more patients – we get through the patients quicker having Nurse Practitioners here and often we don’t have a lot of staff; sometimes we’re down so Nurse Practitioners really help us out” Site 1 ACN
	“it helps with our KPIs^*^ because they’re seeing patients quicker … we get a lot of [low acuity patients] so they can fast track a lot of patients. And that helps clear the waiting room; gets patients in and out quickly” Site 2 NUM
	“at the moment we’ve got small numbers of people, they’re available for part of the day and they see a particular set of patients. I think, you know, in the UK where there’s a large number of people, they run an entire section of the department and they’re responsible for that entire group of patients, I could see that might be of benefit … it’s a bit tokenistic [here] at the moment, you know, we have a nurse practitioner, or two nurse practitioners in a department where we’ve got, I don’t know, two hundred and thirty staff in the department and we’ve got two nurse practitioners so it’s still such a tiny fraction of our population … I think if I take a global view, the overall impact is relatively small.” Site 2 ED Director
	“Emergency Departments offer a lot more services for patients. We’re a bit of a one stop shop and we offer things where there are gaps so the gap might be related to time so “at ten o’clock at night where do I go with my problem, there’s not a GP” or “I’ve cut my hand and I need to have stitches and my GP doesn’t do stitches so where do I go?” so you come to the ED. I’ve broken my bone and my GP doesn’t put plasters on so where do I go? So the nature of ED and the service that we provide [has changed] … there’s a gap in service provision so you know the doctors were just seeing lots and lots of people coming through and you know one asks themself does it make good sense to have a person that’s got a cut leg that needs half a dozen stitches put in their leg, does that make sense to take a doctor away from a patient with high complex medical problems to stitch up a person with a leg. It certainly needs an advanced level of clinical thinking to examine the leg and make sure that there’s no underlying structures involved and that there’s no nerve damage or tendon or whatever so … it makes sense to have nurses who are trained and have these skills and ability to do that type of stuff, to do those sorts of things so the doctors can be freed and do other things” Site 1 NP
	“they appear to have taken a second line role rather than a front line role in terms of patient care. Nurse practitioners would be rostered to a shift but they will base themselves, for significant portions of the shift, in an office area away from the floor and so they will come back down when they see or feel that there’s work to be done but it’s – they’re not there at the front line…it’s a nursing position so they’re still primarily responsible to the nurse manager of the department. They have clinical responsibilities to myself and to the other staff specialists because we are their clinical supervisors but their management line is the nurse manager … I think it’s going to require some intervention to get that to happen more the way I would like to see it” Site 2 ED Director
	“yesterday, for example around eight o’clock the staff specialist gives me a call and says, “Look, the nurse practitioner was in the office til eight o’clock” and not a single patient was seen … I’m not very happy about it but administratively they come under the Director of Nursing in this hospital” Site 1 ED Director
	“I think they’re actually going to hit an interesting crossroad because I think one of the big drivers for the role was the fact that there was a need and the need was for having more of decision makers and there was a relative shortage of junior doctors who were the traditional people filling that role, that’s the role that they have basically stepped into. But with university placement planning and all the rest we’re now actually hitting in fact what many people are describing an excess of junior doctors coming through … and so now there’s going to be a flood of these doctors. I think that’s going to threaten the viability of the nurse practitioner program because the hospitals are going to have to employ these people and I wonder if they’re going to have any interest in pursuing this alternate group of practitioners” Site 2 ED Director
Impact on other Clinical Roles	“they’ve freed the doctors that work in the Subacute area from seeing those patients to go on and see patients that are a little higher acuity” Site 1 Doctor
	“the ones that we have have worked very well and they would definitely have up-skilled nurses around them and empowered them to do things usually otherwise they would not have done” Site 1 Doctor
	“They’re very sharing with their knowledge, they’re great educators to the other nurses. You know, you learn so much from them; you often learn a lot more from them than you do the doctors because they’re willing to sit down and talk to you and take the time to explain things” Site 1 ACN
	“I think role modelling, [NPs have] an opportunity to role model for other nurses starting out or nurses that have gone two thirds through their career and then thought, “Oh, I can do this”… I think other nurses will go along and say, “Well gee, hey I can do that. You know I could commit to that period of study and I could be as autonomous as this person” so that’s a goal so role modelling” Site 2 NUM
	“it has an impact in terms of work relationships, because you’ve got someone who’s a nurse but now is not actually someone who you would task to do something that you would task the other nurses to do because they’re seeing their own patients so that sort of relationship is different” Site 2 ED Director
Impact on Care Delivery	“their interaction with the patient will be similar to the ones that would be from a doctor. Perhaps they might even spend more time and explain better or explain more than a doctor would given that he’s - we might be superficial about some things - they might spend more time talking to them about aftercare and so on because they’ve been through a nursing perspective and have in the past, dealt with patients. If anything these minor patients with these kind of problems might be better dealt with by people with nursing backgrounds than doctors for instance” Site 1 Doctor
	“They bring expertise and continuative care and holistic care” Site 2 NUM
	“the healthcare system will evolve. As it stands today, it’s not going to hold; we need to look for other solutions to how we look after our patients today, and I think that’s where the Nurse Practitioners will take a huge role … I think we just need to have a reform in the way we run the healthcare system - it’s still very hierarchical and it’s still very medically driven and I think we need to look for other solutions – it’s not necessarily medically driven healthcare that we need. I think you need to look at what patients want and need and let patients maybe drive it more; give the power back to the patients and see what they need. They don’t necessarily need a script for antibiotics every time they go to a doctor, they might need to get other things and start changing their lifestyles and get input from social workers and get other people to be [involved] rather than spending hours and hours with a doctor and I think Nurse Practitioners are really good at doing that. Being a nurse, you have a more holistic view – not that doctors don’t do that, but I think we do have more insight into other professions … they often use the doctor as the gold standard and my question would be, is that the gold standard? Why do you compare us to a doctor? It might not be the gold standard, [or] it might [be] in something else” Site 1 NP

*Key performance indicators.

ED directors at both sites expressed a significantly different view to that of NPs. ED directors perceived that the number of NPs employed in the ED was statistically insufficient to effect any real improvement to patient flow. Further, whilst their contribution to primary care was acknowledged, one Director questioned the cost-effectiveness of NPs in the time-sensitive ED environment where he perceived NPs could not match the patient throughput achieved by a junior doctor despite the greater cost of such positions. ED directors also observed on occasions the non-clinical aspect of the NP role preventing NPs from taking a “frontline” approach to care. Specifically, directors reported that NPs may spend considerable time in an office when the ED floor is especially busy. Directors identified this as a governance issue as they were not administratively responsible for the NPs and were excluded from negotiating the mix of NP responsibilities which were under the governance of the Director of Nursing. However, it was conceded that whilst junior doctors were largely transient, NPs contributed to increasing the pool of permanent staff in the ED. Further, Directors at both sites questioned the future of NPs in metropolitan EDs in the advent of an anticipated influx of medical graduates in Australia.

### Impact on other clinical roles

Senior doctors and nurses agreed that the NP role had changed the task mix of doctors in relieving their subacute workload (Table 3). NPs and nurses also felt that they contributed to the upskilling and empowerment of nurses through their roles as educators. Senior nurses felt that NPs served as role models and played a large part in exposing other nurses to an advanced clinical pathway in addition to the traditional areas of management and education. One senior nurse felt that she had learnt more from NPs who were more willing to take time to explain and clarify complex concepts. The ED director from one hospital described the NP as “nurses who practice in doctors’ clothing” and felt that they challenged the traditional nurse-doctor work dynamic. One NP observed a subtle attitude change in certain doctors as they adjusted to collaboration with a relatively autonomous nurse.

### Impact on care delivery

Senior doctors, nurses and NPs agreed that NPs embraced a more holistic model of patient care (Table 3). Senior nurses felt that NPs provided a level of service which supplemented the medical expertise of a doctor with the caring background of a nurse. Senior doctors agreed that the nursing background of NPs meant that they were more likely to spend time explaining the disease and care processes and were more mindful of the non-clinical attributes of a patient likely to affect aftercare outcomes such as their socioeconomic circumstances.

NPs felt that they were part of a prominent shift in clinical decision-making from the clinician to the patient and an increased emphasis on preventative health and health promotion, advocating patient education and self care. Additionally, NPs reported that they were more facilitative of patient involvement in care-based decision-making and a team-based approach to care that is traditionally dominated by the perspective of a single doctor. NPs expressed frustration at what they felt was a consistent judgement of their practice against doctor-provided, or “gold standard” of care. They argued that the NP model of care rather complemented doctors’ practices to address changes in consumer demands and the nature of care delivered in the contemporary metropolitan ED. One ED Director stressed the importance of adherence to established scopes of practice to recognise that the NP was an independent practitioner who was nevertheless part of a multidisciplinary team.

## Discussion

Our study found that senior doctors and nurses, and NPs expressed differing views on the impact of NPs on care delivery. NUMs were positive about NPs’ effects on ED functioning, believing that they improved patient throughput and contributed to alleviation of patient waiting times. NPs felt that they were ideal candidates for addressing the shifting nature of the ED from a purely acute care setting to an increasingly subacute treatment source. In contrast, ED directors questioned the ability of a very small number of NPs to deliver efficiencies which resulted in tangible improvements. Further, ED directors reported observing NPs prioritising office-based, non-clinical tasks over frontline care delivery during busy shifts.

A shift in the subacute workload from doctors to NPs was observed by NPs, senior doctors and nurses. Nurses and NPs reported a positive NP influence on their nursing peers in the form of empowerment, education and role modelling for advanced practice career pathways. All respondents agreed that NPs adhered to a more holistic care paradigm whilst NPs challenged the validity of doctor-provided care as the established “gold standard” of medicine over the merits of consumer empowerment and a preventative care paradigm.

Studies have quantified clinician perceptions and understandings of the NP role when evaluating NP implementation and service delivery through structured surveys [25,26]. Whilst the NP role is generally viewed favourably by both medical and nursing staff, gaps were identified regarding staff knowledge about the functions of an NP, such as the scope of practice and clinical practice guidelines. In-depth, qualitative studies which give insight to the reasons for such views and the impact NPs have on care delivery have been limited. Findings from our study identified two key themes which should be addressed to facilitate role acceptance: conflicting care paradigms and conflict of governance.

### Conflicting care paradigms

Adopting a macro view in their assessment, physician ED directors questioned the measurable impact of NPs as they are such a small portion of a large total ED workforce. Another concern they had was the cost-effectiveness and validity of the relatively more time-consuming preventative model of care adopted by NPs in a highly time-sensitive care setting where focus was on expedient patient movement through the department. However, drawing from a front-line perspective, senior nurses perceived NPs as pivotal in maintaining consistent patient flow through the department and relieving extensive wait times for the increasing numbers of low-acuity ED presentations. A number of studies have shown that NPs and doctors have similar levels of health outcomes and resource use [9,27-36], and therefore in our study, senior nurses’ views are supported by the existing literature. Studies have also shown that the impact of NPs in EDs specifically have been positive, showing improvements in key performance indicators [9,31,32,37-39] such as reduced patient waiting times, length of stay, cost effectiveness and patient satisfaction [9,39,40].

NPs perceived the contribution of NP practice lay in its accommodation of both the changing nature of consumer demand on the contemporary ED, as well as a general shift in care paradigm within the broader healthcare environment. In addition to the traditional role of providing urgent and life-saving treatment, the emergency care system now provides primary care services to an increasing number of patients who cannot access alternative services [41]. Changes in consumer health trends owing to a rapidly ageing population have also shifted care delivery paradigms [5,6]. The shift involves moving from an emphasis on a curative paradigm of care delivery focussed on disease treatment to a preventative care paradigm espousing increased patient involvement, multidisciplinary team-based decision-making and health education [5,6]. Access block, medical workforce shortages and increased demand on EDs have long seen NPs being advocated as a potential solution [42]. The conflicting care paradigms between doctors and nurses in our study, relating to the role of the NP, need to be addressed to achieve best use of NPs in the ED [18].

### Conflict of governance

ED directors from both sites observed that, at times during periods of peak patient flow, NPs were removed from front-line clinical work by the non-clinical aspect of their role. Whilst NPs practised autonomous management of presentations within specific scopes of practice and were clinically responsible to the ED director, the Director of Nursing retained administrative governance over the NP position and determined task mix. Duality of NP governance appears reflective of the ambiguous position of the NP’s role between nursing and medicine. These ambiguities are best addressed through dialogue and collaboration between relevant medical and nursing representatives, as conflicting demands and priorities from multiple sources of management can create problems [43].

### Limitations

Discrepancies in the role and function of NPs across sites and States limit generalisability of results beyond the study sites. The small sample of NPs is also reflective of the still limited availability of NPs. As our intention was to undertake in-depth interviews, the total number of interviews was modest. A larger sample of staff working with NPs in a future study would be important in validating our findings.

## Conclusions

The impact of the NP role in the ED was perceived differently by different groups of participants. The results demonstrate a misalignment of frames with implications for further development of the NP role. Reconciliation of perceptions of the role of the NP is essential to ensure that they achieve their desired goal of improving patient outcomes and efficiencies within the ED.

## Competing interests

The authors declare that they have no competing interests.

## Authors’ contributions

JL, JW, JC and AG contributed to the conceptualisation and design of the study. JL undertook data collection, analysis, and drafting of the manuscript. JW, JC, AG and JB contributed to results interpretation and critical revision of the manuscript. All authors read and approved the final manuscript.

## Pre-publication history

The pre-publication history for this paper can be accessed here:

http://www.biomedcentral.com/1472-6963/13/356/prepub
